# Atypical Presentation of Amyloidosis in a Female Patient with Muscle Weakness

**DOI:** 10.1155/2023/1553163

**Published:** 2023-04-13

**Authors:** Raziyeh Lashkari, Maryam Loghman, Leila Aghaghazvini, Hiva Saffar, Bentolhoda Ziaadini, Reza Shahriarirad, Mohammad Nekooeian, Mohammad Nejadhosseinian, Majid Alikhani

**Affiliations:** ^1^Semnan University of Medical Sciences, Semnan, Iran; ^2^Department of Internal Medicine, School of Medicine, Rheumatology Research Center, Shariati Hospital, Tehran University of Medical Sciences, Tehran, Iran; ^3^Department of Radiology, Shariati Hospital, Tehran University of Medical Sciences, Tehran, Iran; ^4^Department of Pathology, Shariati Hospital, Tehran University of Medical Sciences, Tehran, Iran; ^5^Neurology Research Center, Kerman University of Medical Sciences, Kerman, Iran; ^6^School of Medicine, Shiraz University of Medical Sciences, Shiraz, Iran; ^7^Thoracic and Vascular Surgery Research Center, Shiraz University of Medical Sciences, Shiraz, Iran; ^8^Health and System Research Center, Shiraz University of Medical Sciences, Shiraz, Iran; ^9^Shiraz Nephro-Urology Research Center, Shiraz University of Medical Sciences, Shiraz, Iran; ^10^Joint Reconstruction Research Center, Tehran University of Medical Sciences, Tehran, Iran; ^11^Rheumatology Research Center, Tehran University of Medical Sciences, Tehran, Iran

## Abstract

Muscle involvement represents a well-recognized but rare manifestation of amyloidosis. Here, we report a 40-year-old female who presented with muscle weakness, musculoskeletal pain, and proteinuria, which was eventually diagnosed as myopathic amyloidosis based on muscle biopsy results. A multidisciplinary approach appears to be the cornerstone of the diagnostic work up for recognizing the unusual amyloid myopathy.

## 1. Introduction

In primary systemic amyloidosis, insoluble immunoglobulin light chains clump together to create amyloid fibrils. In about 90% of patients, a monoclonal protein is traceable in the urine or serum [1]. The most common kind of acquired amyloidosis is immunoglobulin light chain (AL) amyloidosis. The condition is caused following a misfolded monoclonal light chain generated by a plasma cell or B-cell clone, which has a proclivity for aggregation and tissue deposition, resulting in organ failure. Although it is primarily a systemic illness with the potential for widespread organ harm, localized deposits have been documented [2]. Muscle involvement has been rarely reported in AL amyloidosis, and amyloid myopathy is frequently misdiagnosed and delayed. Amyloid myopathy is a symptom that can occur before the diagnosis of systemic AL amyloidosis. Herein, we describe a case of a 40-year-old female who presented with proteinuria, muscle weakness, and musculoskeletal pain, which was eventually diagnosed as myopathic amyloidosis.

## 2. Case Report

### 2.1. Current Presentation

Our patient is a 40-year-old woman hospitalized with progressively worsening muscular weakness as her chief complaint since three months prior to hospitalization. The patient also complained of difficulties rising from a seated posture and proximal muscle pain. Shortly after that, she developed skin stiffness, tightness of the palms and soles, muscular stiffness, and pain in the left knee. In addition to worsening her existing symptoms, she presented with widespread body pain, wrist and ankle arthralgia, morning stiffness, tingling, and sole paresthesia around one month ago. Moreover, she reported discomfort in the temporomandibular joint and difficulties opening her mouth because of TMJ pain. She also complained of weight loss, night sweats, and dysphagia beginning two weeks before her admission, and these symptoms progressed in parallel with her other symptoms.

### 2.2. Past Medical History

Her problem started about three years ago when she was found to have mild hematuria and proteinuria on routine examinations. At that time, proteinuria was mild, and the initial laboratory tests such as fasting blood sugar, HbA1c, hepatitis B and C serology, antinuclear antibodies (ANA), anti-double-stranded DNA (anti-dsDNA), C3, C4, anti-glomerular basement membrane (anti-GBM), and antineutrophil cytoplasmic antibodies (ANCA) were all unremarkable. However, due to an increase in proteinuria and a kidney biopsy demonstrating focal mild mesangial proliferation but negative immunofluorescent results, prednisolone and cyclosporine were administered. The patient was then closely followed by a nephrologist. During that time (around one and a half year before current hospitalization), serum protein electrophoresis was requested which demonstrated albumin: 68.4% (normal: 55.8–66.1%), alpha one: 4.6 (normal: 2.9–4.9), alpha 2: 9.6 (normal: 7.1–11.8), beta 1: 4.8 (normal: 4.7–7.2), beta 2: 4.1 (normal: 3.2–6.5), gamma: 8.5 (normal: 11.1–18.8), total protein: 6.9 g/dl, and A/G ratio: 2.16. Urine electrophoresis demonstrated normal range of protein factors. Since regular urine protein measurements had a downward trend, cyclosporine was discontinued, and prednisolone dosage was slowly tapered to 5 mg daily. Other medical history included peptic ulcer disease, nonalcoholic fatty liver disease, and hypothyroidism, controlled with levothyroxine and proton-pump inhibitors.

### 2.3. Physical Examination

**Figure 1:**
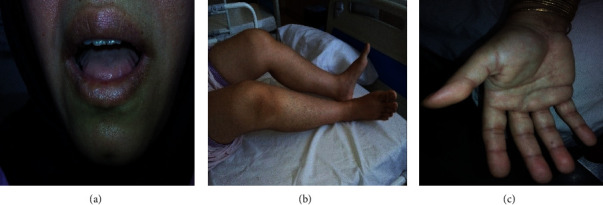
(a) Macroglossia and lateral scalloping of the tongue, (b) flexion contracture of the knee joint, and (c) skin stiffness of palms and sclerodactyly.

Physical examination showed normal vital signs. Macroglossia and lateral tongue scalloping were noted while the patient was unable to protrude her tongue (Figure 1(a)). The findings of extremity examination consisted of fascia hardening of soles and palms, sclerodactyly (Figure 1(c)), arthritis of both wrists and right ankle, lower extremity pitting edema, and muscle stiffness which was particularly evident in biceps and triceps. Furthermore, flexion contracture of knees (Figure 1(b)), elbows, shoulders, and hip was found. Even though the muscle power was 5/5 in all extremities, the squatting test was impaired. There were no signs of neuropathy in physical examination.

### 2.4. Investigations

**Figure 2:**
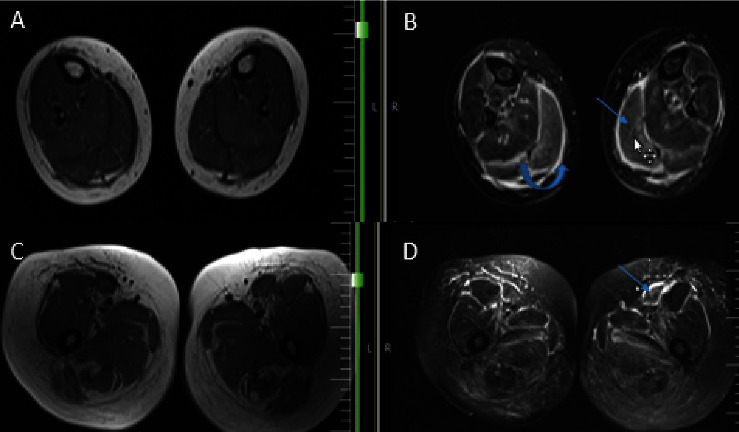
(a and b): MRI of both legs; T1W (a) and fluid sensitive fat sat (b) sequences; perimuscular and fascial edema (curved arrow) and intra muscular edema (arrow) within gastrocnemius muscles; (c and d): MRI of both thighs T1W (c) and fluid sensitive fat sat (d) sequences; perimuscular and fascial edema (arrow) more in anterior compartment.

**Figure 3:**
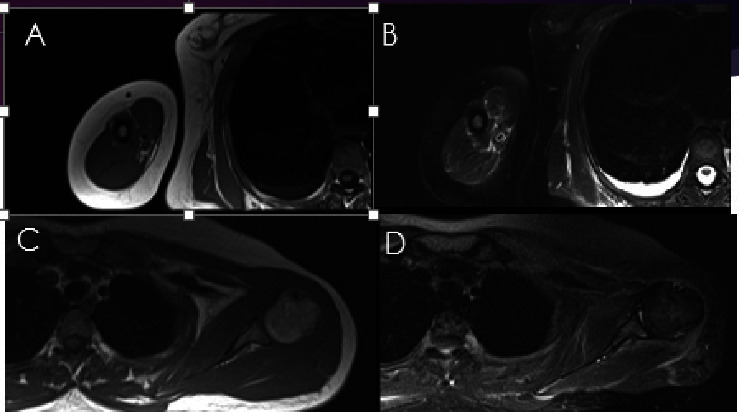
MRI of right arm T1W (a) and fat sat (b) and (c)-(d) MRI of left shoulder T1W (c) and fat sat (d) sequences. Note to the diffuse perimuscular and fascial edema more along the latissimus dorsi and rotator cuff muscles (arrows).

Given that the patient had proteinuria, a nephrologist requested laboratory tests, including fasting blood sugar, HbA1c, hepatitis B and C serology, ANA, anti-dsDNA, C3, C4, anti-GBM, and ANCA, which were all normal. All kidney function tests were unremarkable; however, previous kidney biopsy showed focal mild mesangial proliferation. In addition, the immunofluorescent study of kidney tissue was negative for IgG, IgA, IgM, C3, C4, C1q, fibrinogen, kappa, and lambda. Due to the recent addition of proximal muscle weakness, skin thickening, and arthralgia to the preexisting proteinuria, she was hospitalized, and additional laboratory tests regarding the diagnosis of myositis due to connective tissue diseases were requested, with all serological results being negative. Myopathy immunologic panel testing, scleroderma-related antibodies, and thyroid function test also demonstrated normal results. Furthermore, muscle enzyme results, including lactate dehydrogenase, creatine phosphokinase, and aldolase, were unremarkable. Electromyography and nerve conduction velocity (EMG-NCV) study suggested a mild myopathic process. For bilateral thigh and arms, magnetic resonance imaging (MRI) revealed widespread perimuscular fluid and edema with concomitant subcutaneous fluid around muscles, as well as grade 1-2 fatty changes in all pelvis and leg and arm muscles along with mild to moderate intramuscular edema (Figures 2(a)–2(d)). Another finding of upper extremity MRI was bilaterally diffuse subcutaneous fat thickening (Figures 3(a)–3(d)). There was no evidence of bone lesions.

Given the concurrent occurrence of macroglossia and proteinuria, amyloidosis was not an unlikely diagnosis at the time. However, the likelihood of amyloidosis caused by an underlying plasma cell dyscrasia increased when the patient's laboratory results revealed high calcium levels, an elevated erythrocyte sedimentation rate (ESR), and anemia. Serum electrophoresis measurements were alpha 1: 7.4%, alpha 2: 14.9%, beta 1: 5.4%, beta 2: 4.7%, and gamma: 7.9%; however, in serum immune typing electrophoresis, despite markedly decreased free kappa to free lambda ratio, no obvious monoclonal band was detected. The urine electrophoresis pattern was in agreement with a mixed tubular and nonselective glomerular proteinuria. Moreover, urine immune fixation electrophoresis showed two precipitant lines with lambda and kappa. In revaluation of previous kidney core needle biopsy slides, no amyloid deposit on light microscopy was identified. Kidney biopsy revealed no evidence of amyloid deposit within the glomeruli and in the wall of tubules and blood vessels based on H&E staining. Abdominal wall fat biopsy showed no significant pathologic changes, and Congo red staining was negative for amyloid deposition.

**Figure 4:**
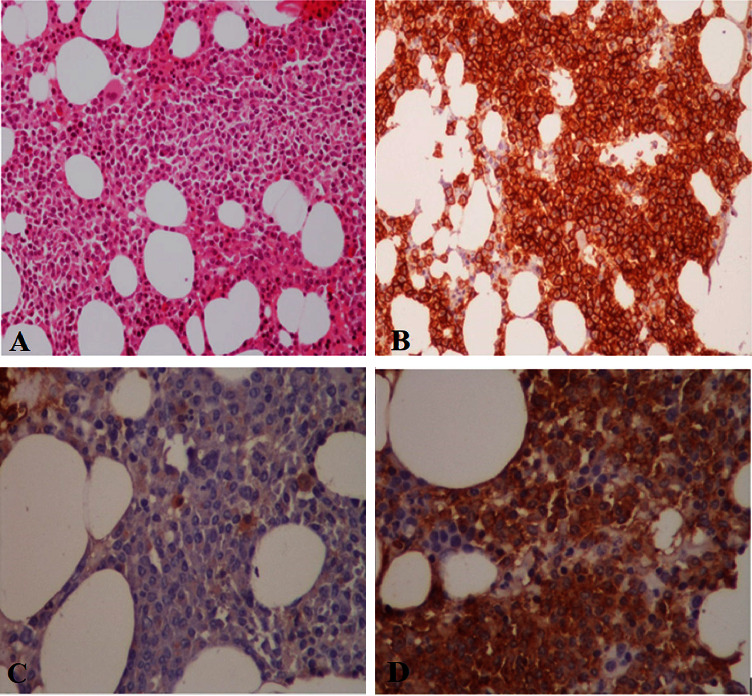
Bone marrow shows sheets of plasmacytoid cells. (a) H&E. (b) CD138. (c) Lambda. (d) Kappa.

**Figure 5:**
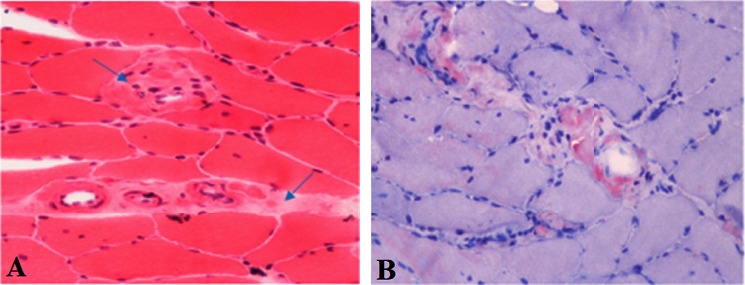
Muscle biopsy of the medial head of the gastrocnemius muscle demonstrating deposition of hyalinized amorphous extracellular eosinophilic material in vessel walls and endomysial connective tissue. (a) Hematoxylin and eosin. (b) Congo red.

In order to establish the diagnosis of plasma cell dyscrasia, a bone marrow sample and histologic examination demonstrated the replacement of marrow spaces by sheets of plasmacytoid cells. The majority of cells were positive for CD138 with lambda light chain restriction (Figure 4). Biopsies were taken from the medial head of the gastrocnemius muscle and its fascia to back up the diagnosis. The histologic exam revealed skeletal muscle fibers with variation in size and degenerative/regenerative changes. Vascular structures showed thickening of the wall with deposition of hyalinized eosinophilic material. Also, endomysial and perimysial connective tissue was expanded with foci of deposition of the same material, which was confirmed to be amyloid by Congo red staining (Figure 5). The findings were consistent with amyloid myopathy. As a result, the diagnosis of amyloidosis due to underlying plasma cell dyscrasia with lambda light chain restriction was made. Given the probable involvement of several organs by amyloidosis, we performed further investigations. Except for a grade 1 diastolic dysfunction, cardiac transthoracic echocardiography was normal. Gastroesophageal endoscopy showed esophagitis and gastric ulcer, which were benign according to tissue diagnosis. The CT scans of the abdomen, pelvis, chest, and liver function tests were likewise normal. Neck, arm, and thigh MRI evaluations were similar to previous report and demonstrated widespread perimuscular fluid and edema with concomitant subcutaneous fluid around muscles, as well as grade 1-2 fatty changes of muscles and mild to moderate intramuscular edema.

### 2.5. Treatment and Follow-Up

The patient was referred to the hematology clinic, where chemotherapy regimen consisting of bortezomib, cyclophosphamide, and dexamethasone was commenced. She also received an autologous bone marrow transplant one year after her diagnosis. Since receiving the transplant around eight months ago, the patient's muscle pain and proteinuria have considerably improved but has remaining difficulties in rising from a seated position and mouth opening.

## 3. Discussion

Lubarsch reported muscle involvement in amyloid in a patient in 1929, and the patient had both interstitial and vascular deposits in the heart and skeletal muscle [3]. Despite this, even in individuals with a known dysproteinemia, amyloidosis remains an uncommon cause of myopathy. In a recent study of 3434 AL amyloidosis patients, 1.5% showed a positive amyloid deposition in muscle biopsy, in which 11 (22%) had amyloid muscle involvement. Myalgia, muscle weakness, macroglossia, jaw claudication, dysphagia, skeletal pseudohypertrophy, and hoarseness are common symptoms in individuals with amyloid myopathy [4, 5]. Our patient had a gradual lower-limb muscular weakness, as well as jaw claudication. Our report was similar to a report by Accardi et al. [6] which reported progressive lower-limb muscle weakness with discomfort and stiffness in the hips and shoulders, as well as jaw claudication, without any biochemical markers of muscle involvement. In their report, based on amyloid deposits in the salivary gland, the final diagnosis was considered as systemic AL amyloidosis along with myopathic involvement. However, because no other symptoms or indicators of systemic AL amyloidosis involvement, such as cardiac failure or renal proteinuria, were evident, the myopathy remained the lone clinical presentation of the disease. This was also supported in a study by Liewluck and Milone among AL amyloidosis muscle involvement patients, with 70% presenting with only myopathy [7]. Among specific clinical aspects, including less frequent CPK elevation and earlier age at onset, AL-systemic amyloidosis-associated myopathy diverges from pure isolated amyloid myopathy induced by other amyloidogenic protein subtypes [7]. Isolated amyloid myopathy is most commonly caused by anoctaminopathy-5 and can be observed in 27% of cases of amyloid myopathy [7]. Detection of systemic involvement in amyloid myopathy may be facilitated with laboratory, clinical, and pathological features [8]. In isolated amyloid myopathy, patients are younger and have higher CK, as expected since the main causes are genetic such as dysferlinopathy and anoctaminopathy. However, in AL-systemic amyloidosis, patients are older with normal or slightly increased CK (<2.5 × upper limit). Our patient demonstrated weight loss, peripheral neuropathy, arthropathy, jaw claudication, kidney involvement, macroglossia, and muscle involvement with normal CK levels, which based on the multisystemic involvement, suggests primary AL-systemic amyloidosis.

The duration between the beginning of the first muscular sign and the diagnosis of amyloidosis in this patient was three months. The median period from the initial illness signs to diagnosis was 23 months in a study among AL amyloidosis patients with positive muscle biopsy [4]. Dissimilar to other amyloid proteins, amyloid deposition in cases of AL amyloidosis can affect various organs, and distinct criteria for evaluation of organ involvement have been published [9, 10]. When amyloid accumulation and the consequent organ damage occur primarily in a less frequent place, including the striated muscle, the diagnostic pitfall may be especially relevant. However, diagnosis delay in amyloidosis is common, as evidenced by a recent patient experience study that found that 37.1% of patients acquired the proper diagnostic formulation during their one year follow-up from their onset of symptoms [11].

The preliminary muscle biopsy failed in detecting amyloid accumulation in 19 of the 79 individuals studied by Chapin et al. [12]. This was owing to a variety of factors, including a “random” biopsy not involving that specific muscle area or the lack of Congo staining [13]. Muchtar et al. observed that a diverse muscle disorder was identified in roughly 40% of the cases prior to the histological review [4], which is consistent with this observation. The hazard of disease exacerbation increases through delay in diagnosis, especially with cardiac involvement, which regulates the prognosis and stage [14–16].

Based on previous reports, three basic clinical presenting types of amyloid myopathy have been documented in the literature [4, 12, 17]. The first group has skeletal pseudohypertrophy with palpable nodules in muscles and an abnormal “wood-like” consistency, which is typically linked with macroglossia. Patients in the second category have muscle weakness, especially proximal, possibly accompanied by atrophy but no other symptoms of amyloid tissue accumulation. Since the diagnosis is challenging and should consider all probable differential diagnoses of myopathy, the “atrophic type” poses a clinical problem [18]. A mixed clinical phenotype is the third group.

Claudication was observed in our study and also four of 12 patients in a report by Gertz and Kyle [17] and has previously been linked to amyloid myopathy [19, 20]. It is caused by amyloid accumulation in the bloodstream, which leads to ischemia and occlusion of tiny blood arteries. In the past, many individuals were misdiagnosed as having giant cell arteritis and treated with heavy doses of corticosteroids.

Muscle fiber damage is a poorly understood pathophysiological process. Fibril deposition is routinely detected on perimysial and endomysial arteries in muscle amyloidosis, as in our instance, suggesting prolonged ischemia resulting from endothelial injury as a plausible cause [21]. Denervation atrophy and also necrotic fibers with regeneration signals are less commonly documented [4, 12]. Delaporte et al. found a rise in the synthesis of protein and also cell fusion of human myoblasts in culture following exposure to pure kappa chain serum from a case of muscular pseudohypertrophy [22]. The large interpatient diversity in protein structure and tissue tropism, previously discovered in amyloid proteins produced from similar variable light chain gene, is among the noteworthy features of AL amyloidosis. The specificity along with the kind of organ damage can be dramatically influenced by a small alteration in the amino acid sequence acquired during hypersomatic mutation of immunoglobulin genes [23].

## 4. Conclusion

We presented a case of primary systemic amyloidosis with multisystemic involvement. A multidisciplinary approach appears to be the foundation of the diagnostic work up for recognizing unusual amyloid myopathy. Muscle biopsy, identification of fibril type, confirmation of amyloid deposition, assessment of underlying amyloidogenic condition, and assessment of the severity and amount of amyloidotic organ involvement are all essential steps in diagnosing and staging this disorder.

## Data Availability

The data used to support the findings of this study are included within the article and are available from the corresponding author upon request.
